# Supplementary material for: Epidemiological hypothesis testing using a phylogeographic and phylodynamic framework
Source: Nat Commun. 2020 Nov 6;11:5620. doi: 10.1038/s41467-020-19122-z (PMC7648063; doi:10.1038/s41467-020-19122-z)
Supplement: Supplementary file 1 — Supplementary Information [file 41467_2020_19122_MOESM1_ESM.pdf]

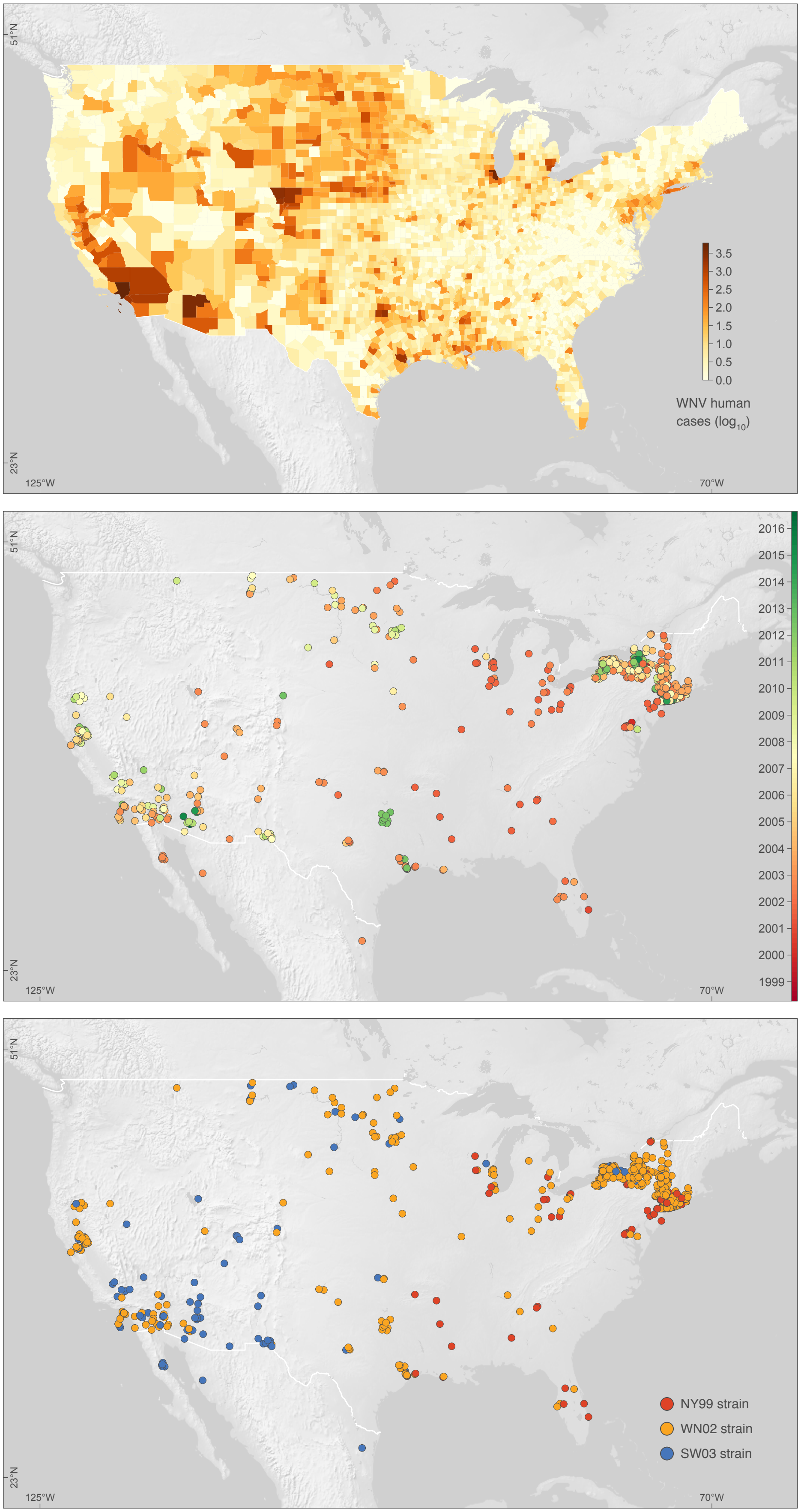

Figure S1. Distribution of human cases of West Nile fever and sampling maps of WNV genomes analysed in the present study. We first report the log_{10}-transformed number of reported human cases per US county (source: Centers for Disease Control and Prevention, `www.cdc.gov/westnile`). In the first sampling map, sample positions are coloured according to sampling times and in the second sampling map, sample positions are coloured according to three phenotypically relevant genotypes (NY99, WN02 and SW03). These genotypes were identified on the basis of the WNV database published on Nextstrain (`https://nextstrain.org/WNV/NA?c=lineage`).

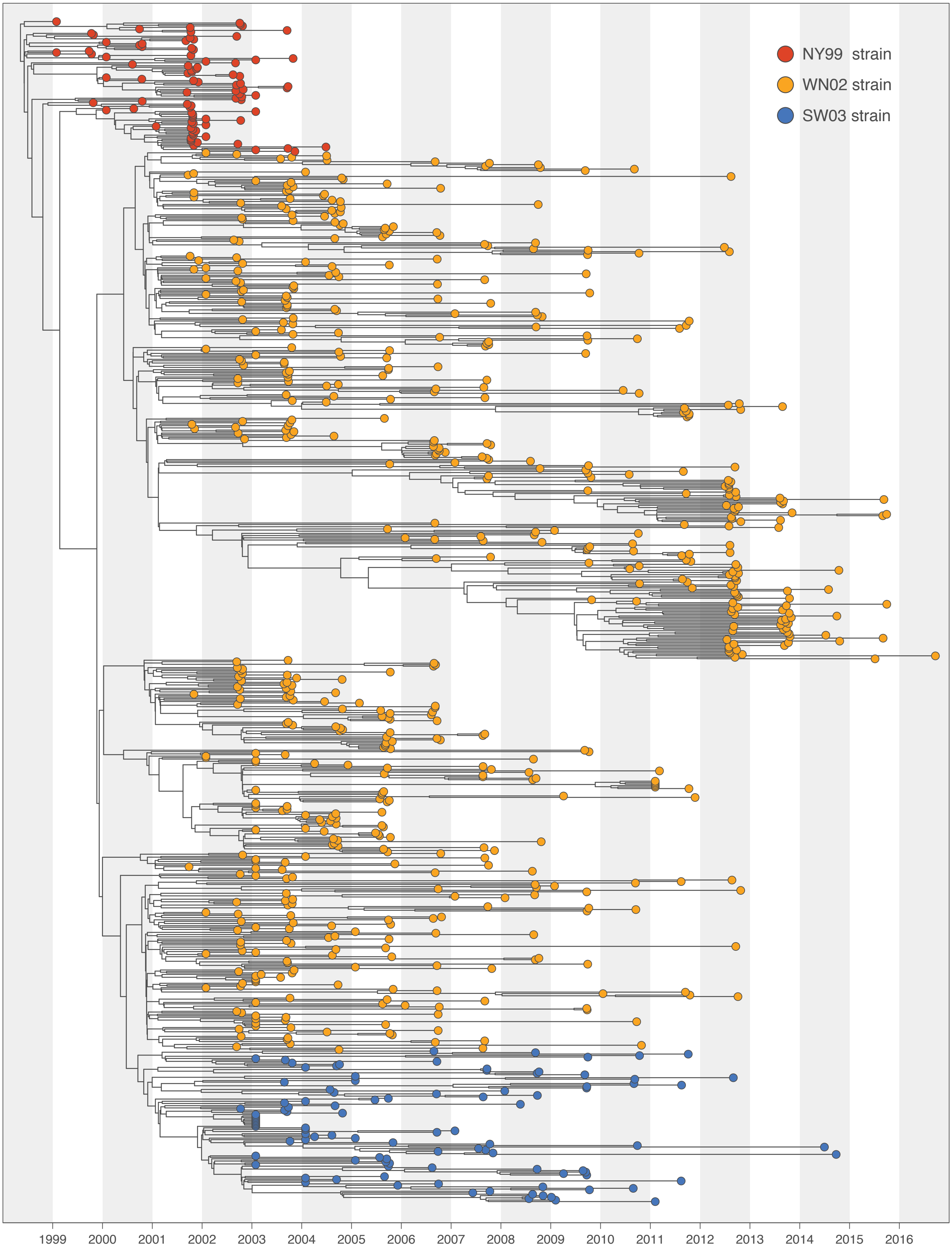

Figure S2. Time-scaled maximum clade consensus (MCC) tree obtained from the phylogenetic analysis of WNV genomes sampled in North America. Tip nodes are here coloured according to three phenotypically relevant genotypes (NY99, WN02 and SW03). These genotypes were identified on the basis of the WNV database published on Nextstrain (`https://nextstrain.org/WNV/NA?c=lineage`).

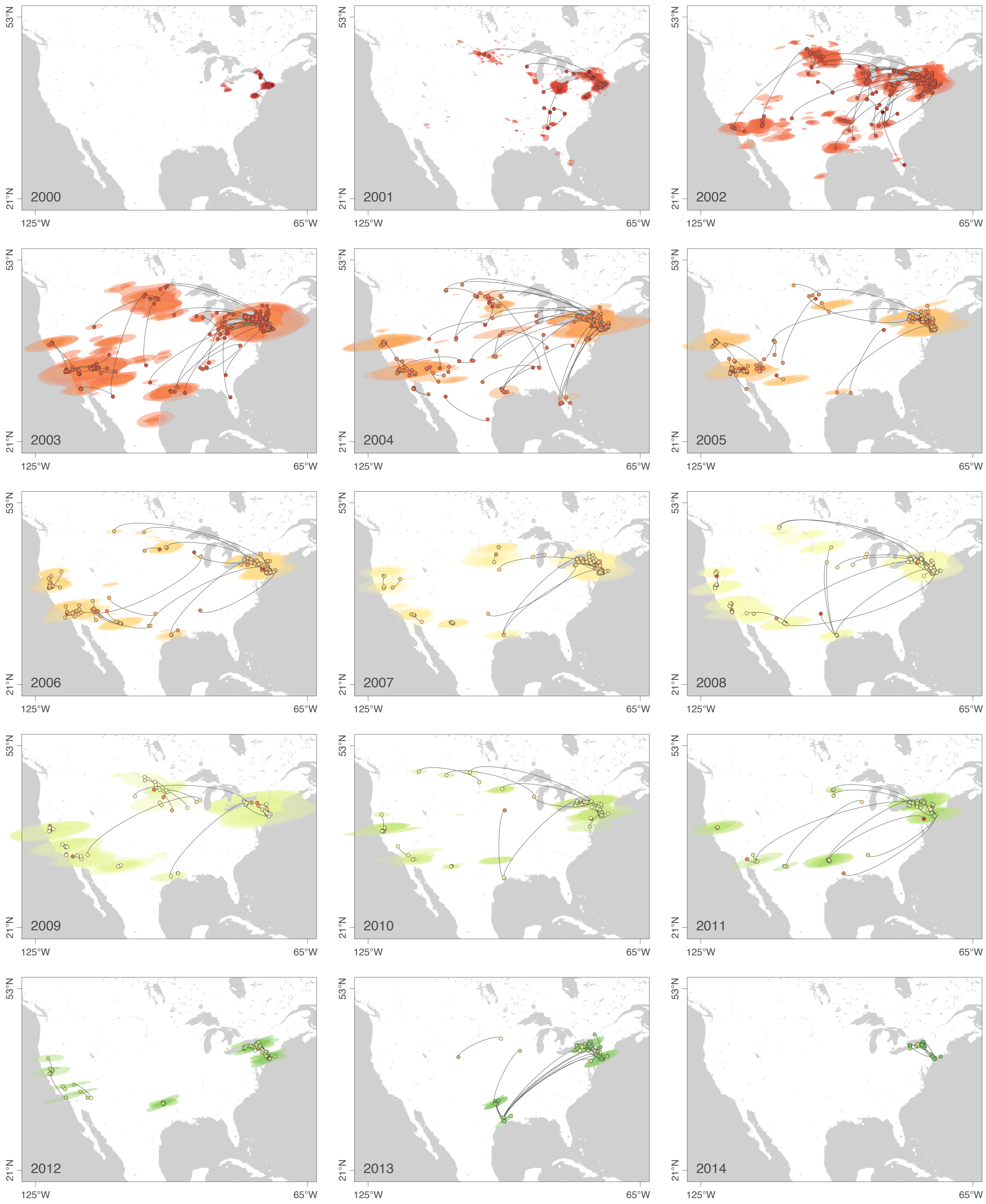

Figure S3. Snapshots of WNV lineages dispersal history in North America from 2000 to 2014. Dispersal history of WNV lineages was inferred with a continuous phylogeographic approach based on 100 posterior trees (see the text for further details). Each map corresponds to a specific year and displays the MCC (maximum clade credibility) tree branches whose youngest ending nodes occurred during the considered year. These MCC trees are also superimposed on 95% HPD reflecting phylogeographic uncertainty. Nodes of the trees, as well as HPD regions, are coloured from red (the time to the most recent common ancestor, TMRCA) to green (most recent sampling time), which corresponds to the colour scale used in Figure 1.

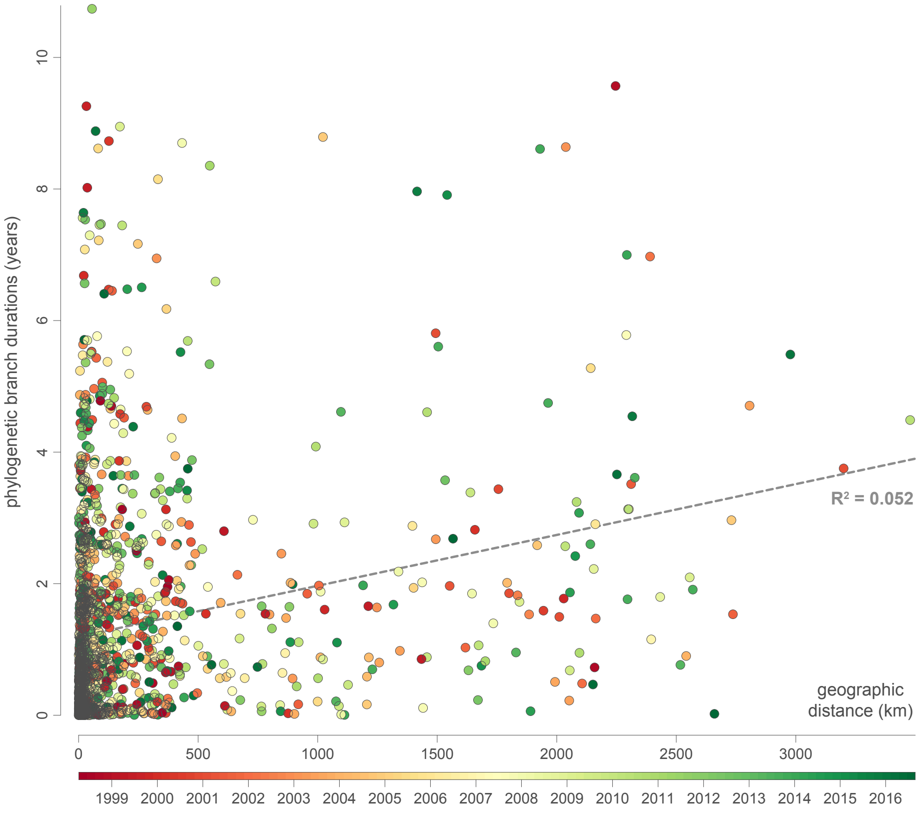

Figure S4. Comparison between the maximum clade credibility (MCC) tree branch durations and geographic distances travelled by these MCC tree branches. Each dot corresponds to a distinct MCC tree branch and is coloured according to the time of occurrence of the youngest node of that branch. We also report the coefficient of determination (R^2) obtained from the linear regression between branch durations and geographic distances. This scatterplot highlights the occurrence of both relatively slow and relatively fast long-distance dispersal events associated with MCC tree branches, which is also reflected by the low correlation estimated between branch durations and geographic distances travelled by those branches (coefficient of determination R^2 = 5%).

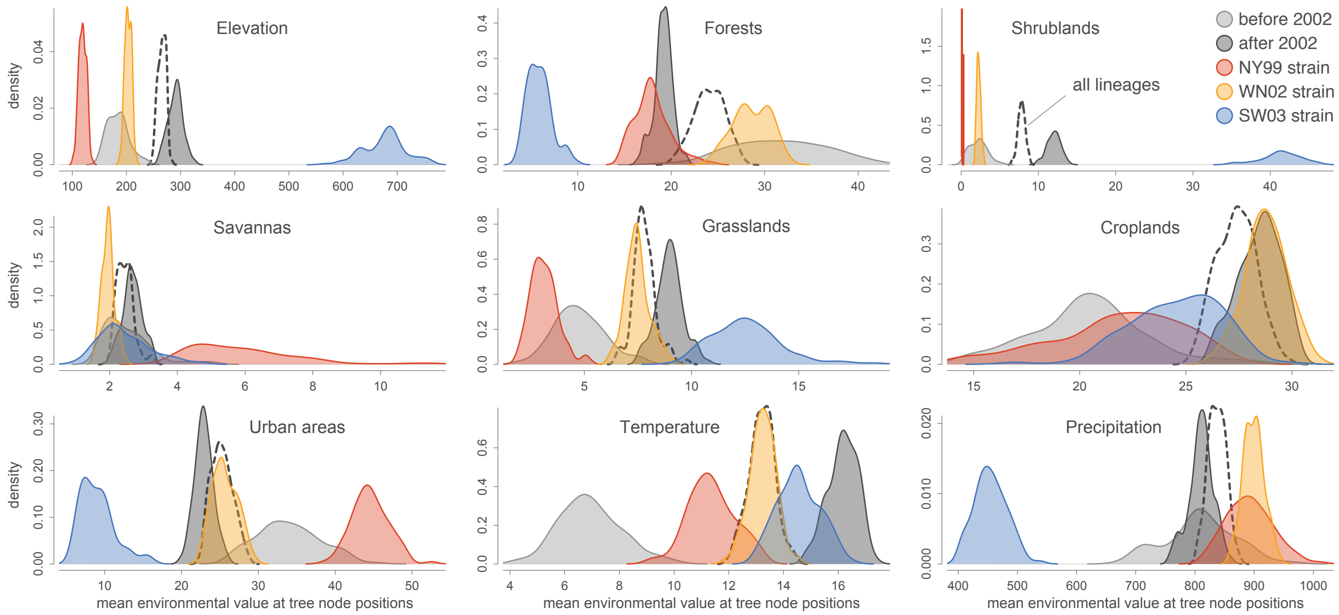

Figure S5. Environmental conditions explored by viral lineages: posterior distributions of the mean environmental values extracted at the tree node positions (i.e. statistic *E*).

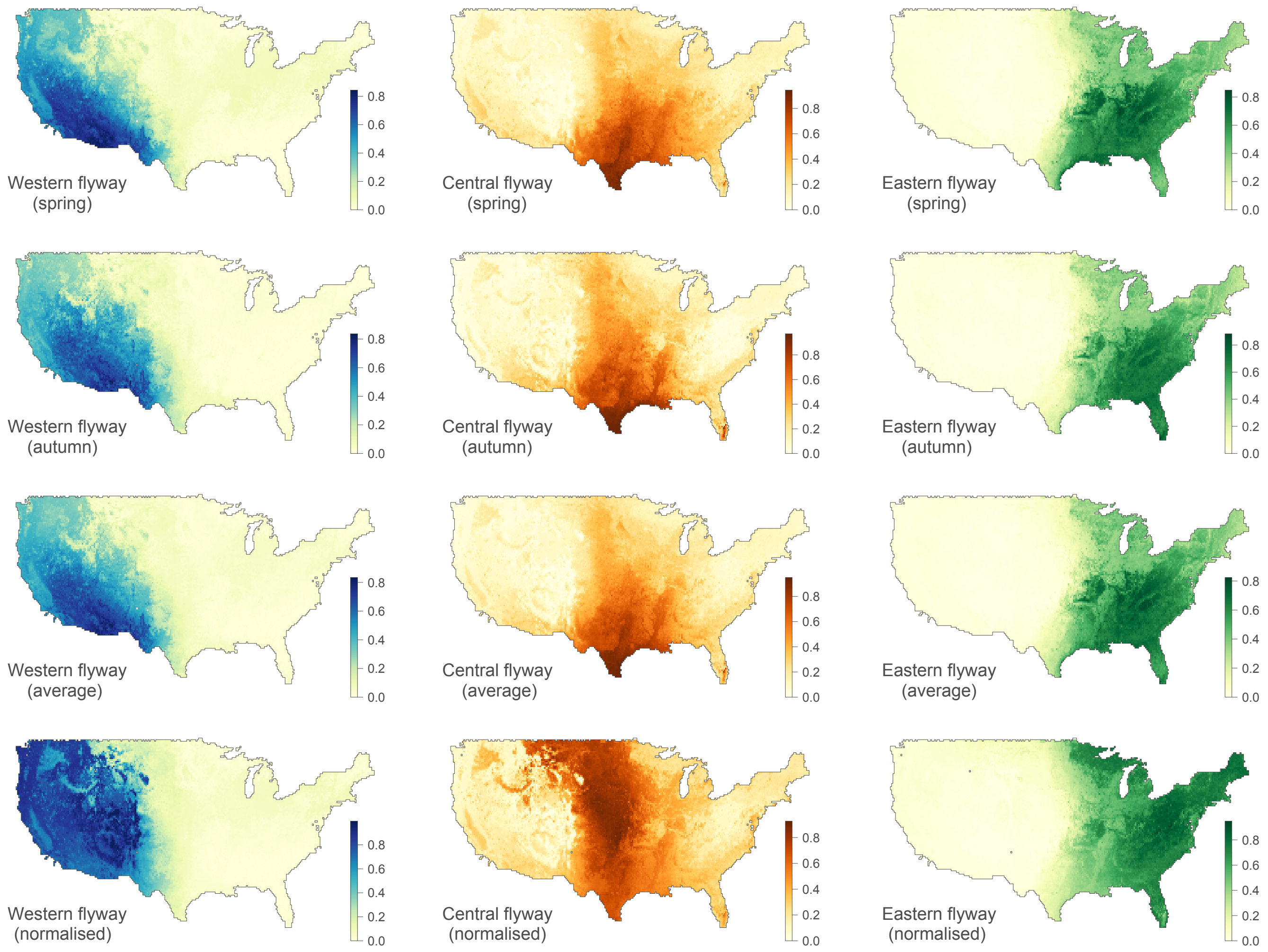

Figure S6. Western, Eastern and Central migratory flyways estimated by La Sorte *et al*. (2014) for terrestrial birds species. The colour scales reflect the likelihood that the studied species are in migration during spring or autumn (see La Sorte *et al*. for further details). Here, we also report maps averaged between spring and autumn, as well as normalised versions obtained by dividing each raster cell by the sum of the values assigned to the same cell in the three averaged rasters. The resulting normalised rasters were here used for testing the association between the migratory flyways estimated for terrestrial birds and the dispersal history of WNV lineages.

Table S1. Source of data for each environmental raster. The topographic raster is only used as background in Figures 1, 3, and S1.

Original raster	Source	URL
Topographic raster	Natural Earth database (here: terrain depicted in shades of grey)	naturalearthdata.com
Elevation raster	SRTM (Shuttle Radar Topography Mission), DEMs (Digital Elevation Models)	webmap.ornl.gov
Land cover raster	IGBP (International Geosphere Biosphere Programme) – categorical raster	www.igbp.net
Annual mean temperature	WorldClim database, version 2.0 (bioclimatic variable "bio1")	worldclim.org
Annual Precipitation	WorldClim database, version 2.0 (bioclimatic variable "bio12")	worldclim.org
Time-series temperature rasters	Interpolated from data of the US National Oceanic and Atmospheric Administration	data.nodc.noaa.gov
Time-series precipitation rasters	Interpolated from data of the US National Oceanic and Atmospheric Administration	data.nodc.noaa.gov
Vegetation index (NDVI)	Normalised Difference Vegetation Index (NDVI), NASA Earth Observation database	neo.sci.gsfc.nasa.gov

Table S2. Impact of several environmental factors on the dispersal location of WNV lineages in North America. We report Bayes factor (BF) supports for the association between environmental values and tree node locations. The results are based on 100 posterior trees obtained by spatially-explicit phylogeographic inference. "A" and "R" indicate if the considered environmental raster was tested as a factor attracting ("A") or repulsing ("R") viral lineages. Following Kass & Raftery (1995), we consider a BF value >20 as strong support for a significant correlation between the environmental distances and dispersal durations (in bold).

Environmental factor	All branches	Internal branches	Before 2002	After 2002	NY99 genotype	WN02 genotype	SW03 genotype
elevation (A)	0.0	0.1	0.1	0.0	0.0	0.0	1.3
elevation (R)	**49.0**	19.0	6.7	**32.3**	**>99**	**>99**	0.8
forests (A)	0.0	0.0	0.1	0.0	0.0	0.0	0.0
forests (R)	**>99**	**>99**	6.7	**>99**	**>99**	**>99**	**>99**
shrublands (A)	**49.0**	**32.3**	5.7	**32.3**	0.4	2.2	**>99**
shrublands (R)	0.0	0.0	0.2	0.0	2.3	0.4	0
savannas (A)	1.1	1.1	6.1	0.9	19.0	1.2	0.3
savannas (R)	0.9	0.9	0.2	1.1	0.1	0.9	3.3
grasslands (A)	0.6	0.4	1.1	0.5	0.4	1.6	0.4
grasslands (R)	1.6	2.3	0.9	1.9	2.7	0.6	2.4
croplands (A)	1.6	1.5	1.7	0.9	1.0	2.7	0.9
croplands (R)	0.6	0.7	0.6	1.1	1.0	0.4	1.2
urban areas (A)	**>99**	**24.0**	4.0	**>99**	**>99**	**>99**	**49**
urban areas (R)	0.0	0.0	0.2	0.0	0.0	0.0	0.0
temperature (A)	**49**	**24.0**	1.0	**49**	6.1	6.7	15.7
temperature (R)	0.0	0.0	1.0	0.0	0.2	0.1	0.1
precipitation (A)	0.0	0.1	0.4	0.0	0.7	0.0	0.0
precipitation (R)	**>99**	8.1	2.8	**>99**	1.5	**32.3**	**>99**

Table S3. Impact of several environmental factors on the dispersal velocity of WNV lineages in North America - analyses based on all phylogenetic branches. The results are based on 100 posterior trees obtained by spatially-explicit phylogeographic inference. "C" and "R" indicate if the considered environmental raster was considered as a conductance ("C") or resistance factor ("R"), and *k* is the rescaling parameter used to transform the initial raster (see the text for further details). For regression coefficients and *Q* values we report both the median estimate and the 95% HPD interval. The Bayes factor (BF) supports are only reported when p(Q > 0) is at least 90%. Following Kass & Raftery (1995), we consider a BF value >20 as strong support for a significant correlation between the environmental distances and dispersal durations (in bold).

Path model	Environmental factor	*k*	Regression coefficient	*Q* statistic	p(Q > 0)	BF
**Least-cost path model**	elevation (C)	10	0.052 [0.034, 0.074]	-0.004 [-0.008, -0.001]	0.02	-
		100	0.049 [0.033, 0.068]	-0.008 [-0.015, -0.001]	0.01	-
		1000	0.042 [0.028, 0.060]	-0.015 [-0.027, -0.006]	0.00	-
	elevation (R)	10	0.052 [0.036, 0.066]	-0.005 [-0.015, 0.000]	0.03	-
		100	0.039 [0.029, 0.050]	-0.019 [-0.036, -0.007]	0.00	-
		1000	0.035 [0.025, 0.045]	-0.023 [-0.041, -0.010]	0.00	-
	forests (C)	10	0.047 [0.031, 0.063]	-0.011 [-0.021, -0.001]	0.01	-
		100	0.037 [0.025, 0.052]	-0.021 [-0.036, -0.008]	0.00	-
		1000	0.032 [0.021, 0.048]	-0.026 [-0.041, -0.012]	0.00	-
	forests (R)	10	0.070 [0.051, 0.095]	0.012 [0.005, 0.021]	1.00	0.3
		100	0.054 [0.038, 0.075]	-0.003 [-0.018, 0.014]	0.32	-
		1000	0.047 [0.032, 0.065]	-0.011 [-0.027, 0.008]	0.12	-
	shrublands (C)	10	0.052 [0.033, 0.070]	-0.005 [-0.009, -0.002]	0.00	-
		100	0.052 [0.033, 0.071]	-0.006 [-0.010, -0.002]	0.00	-
		1000	0.051 [0.034, 0.071]	-0.006 [-0.011, -0.001]	0.01	-
	shrublands (R)	10	0.061 [0.043, 0.084]	0.004 [-0.006, 0.010]	0.72	-
		100	0.028 [0.017, 0.041]	-0.030 [-0.047, -0.013]	0.00	-
		1000	0.018 [0.009, 0.028]	-0.040 [-0.057, -0.021]	0.00	-
	savannas (C)	10	0.060 [0.043, 0.079]	0.002 [-0.003, 0.009]	0.79	-
		100	0.065 [0.047, 0.085]	0.007 [-0.002, 0.015]	0.96	0.3
		1000	0.072 [0.054, 0.095]	0.015 [0.005, 0.023]	1.00	0.4
	savannas (R)	10	0.053 [0.034, 0.073]	-0.005 [-0.008, 0.000]	0.02	-
		100	0.012 [0.007, 0.023]	-0.045 [-0.061, -0.027]	0.00	-
		1000	0.001 [0.000, 0.002]	-0.057 [-0.075, -0.037]	0.00	-
	grasslands (C)	10	0.054 [0.036, 0.077]	-0.003 [-0.007, 0.002]	0.16	-

		100	0.059 [0.042, 0.084]	0.002 [-0.006, 0.009]	0.63	-
		1000	0.058 [0.041, 0.081]	0.000 [-0.016, 0.014]	0.48	-
	grasslands (R)	10	0.041 [0.027, 0.052]	-0.017 [-0.027, -0.009]	0.00	-
		100	0.020 [0.013, 0.027]	-0.037 [-0.055, -0.024]	0.00	-
		1000	0.015 [0.009, 0.022]	-0.042 [-0.061, -0.027]	0.00	-
	croplands (C)	10	0.047 [0.033, 0.063]	-0.009 [-0.022, 0.000]	0.03	-
		100	0.025 [0.016, 0.035]	-0.032 [-0.050, -0.019]	0.00	-
		1000	0.016 [0.008, 0.027]	-0.040 [-0.060, -0.027]	0.00	-
	croplands (R)	10	0.052 [0.034, 0.069]	-0.006 [-0.013, 0.005]	0.08	-
		100	0.039 [0.023, 0.059]	-0.019 [-0.031, 0.000]	0.02	-
		1000	0.035 [0.021, 0.056]	-0.022 [-0.035, -0.004]	0.01	-
	urban area (C)	10	0.058 [0.037, 0.076]	0.000 [-0.003, 0.002]	0.41	-
		100	0.054 [0.036, 0.069]	-0.004 [-0.011, 0.003]	0.13	-
		1000	0.041 [0.027, 0.053]	-0.017 [-0.029, -0.005]	0.00	-
	urban area (R)	10	0.063 [0.042, 0.083]	0.005 [0.002, 0.008]	1.00	1.8
		100	0.052 [0.035, 0.069]	-0.007 [-0.02, 0.011]	0.25	-
		1000	0.014 [0.004, 0.028]	-0.042 [-0.063, -0.018]	0.00	-
	annual mean temperature (C)	10	0.054 [0.037, 0.066]	-0.003 [-0.015, 0.008]	0.25	-
		100	0.055 [0.037, 0.067]	-0.003 [-0.015, 0.010]	0.31	-
		1000	0.055 [0.036, 0.067]	-0.003 [-0.015, 0.010]	0.35	-
	annual mean temperature (R)	10	0.047 [0.034, 0.058]	-0.010 [-0.022, 0.002]	0.04	-
		100	0.042 [0.030, 0.053]	-0.015 [-0.029, -0.003]	0.02	-
		1000	0.041 [0.030, 0.052]	-0.016 [-0.03, -0.003]	0.00	-
	annual precipitation (C)	10	0.051 [0.036, 0.064]	-0.001 [-0.005, 0.002]	0.35	-
		100	0.047 [0.034, 0.060]	-0.005 [-0.011, 0.001]	0.06	-
		1000	0.046 [0.033, 0.058]	-0.007 [-0.013, 0.000]	0.03	-
	annual precipitation (R)	10	0.050 [0.035, 0.064]	-0.002 [-0.005, 0.001]	0.11	-
		100	0.048 [0.034, 0.062]	-0.004 [-0.008, 0.000]	0.04	-
		1000	0.048 [0.034, 0.062]	-0.004 [-0.008, 0.000]	0.04	-
**Circuitscape path model**	elevation (C)	10	0.054 [0.037, 0.066]	-0.003 [-0.015, 0.008]	0.25	-
		100	0.055 [0.037, 0.067]	-0.003 [-0.015, 0.010]	0.31	-
		1000	0.055 [0.036, 0.067]	-0.003 [-0.015, 0.010]	0.35	-
	elevation (R)	10	0.047 [0.034, 0.058]	-0.010 [-0.022, 0.002]	0.04	-
		100	0.042 [0.030, 0.053]	-0.015 [-0.029, -0.003]	0.02	-
		1000	0.041 [0.030, 0.052]	-0.016 [-0.03, -0.003]	0.00	-
	forests (C)	10	0.051 [0.036, 0.064]	-0.001 [-0.005, 0.002]	0.35	-
		100	0.047 [0.034, 0.060]	-0.005 [-0.011, 0.001]	0.06	-
		1000	0.046 [0.033, 0.058]	-0.007 [-0.013, 0.000]	0.03	-
	forests (R)	10	0.050 [0.035, 0.064]	-0.002 [-0.005, 0.001]	0.11	-
		100	0.048 [0.034, 0.062]	-0.004 [-0.008, 0.000]	0.04	-
		1000	0.048 [0.034, 0.062]	-0.004 [-0.008, 0.000]	0.04	-
	shrublands (C)	10	0.063 [0.040, 0.088]	-0.034 [-0.044, -0.026]	0.00	-
		100	0.023 [0.011, 0.039]	-0.071 [-0.096, -0.055]	0.00	-
		1000	0.010 [0.003, 0.021]	-0.086 [-0.112, -0.063]	0.00	-
	shrublands (R)	10	0.101 [0.078, 0.132]	0.006 [-0.009, 0.019]	0.77	-
		100	0.071 [0.050, 0.094]	-0.027 [-0.052, -0.005]	0.01	-
		1000	0.062 [0.043, 0.084]	-0.036 [-0.064, -0.013]	0.00	-
	savannas (C)	10	0.061 [0.044, 0.088]	-0.033 [-0.053, -0.019]	0.00	-
		100	0.028 [0.016, 0.044]	-0.067 [-0.095, -0.045]	0.00	-
		1000	0.019 [0.008, 0.029]	-0.078 [-0.107, -0.054]	0.00	-
	savannas (R)	10	0.077 [0.057, 0.105]	-0.019 [-0.040, 0.000]	0.02	-
		100	0.051 [0.035, 0.073]	-0.045 [-0.073, -0.019]	0.00	-
		1000	0.045 [0.030, 0.065]	-0.052 [-0.079, -0.025]	0.00	-
	grasslands (C)	10	0.086 [0.063, 0.113]	-0.010 [-0.018, -0.005]	0.00	-
		100	0.083 [0.062, 0.111]	-0.012 [-0.022, -0.007]	0.00	-
		1000	0.080 [0.058, 0.107]	-0.016 [-0.027, -0.010]	0.00	-
	grasslands (R)	10	0.035 [0.024, 0.054]	-0.060 [-0.084, -0.041]	0.00	-
		100	0.007 [0.003, 0.015]	-0.089 [-0.117, -0.065]	0.00	-
		1000	0.005 [0.002, 0.011]	-0.091 [-0.12, -0.067]	0.00	-
	croplands (C)	10	0.097 [0.068, 0.127]	-0.002 [-0.007, 0.006]	0.32	-
		100	0.084 [0.058, 0.115]	-0.012 [-0.023, 0.000]	0.01	-
		1000	0.066 [0.041, 0.094]	-0.029 [-0.044, -0.015]	0.00	-
	croplands (R)	10	0.047 [0.032, 0.076]	-0.050 [-0.070, -0.027]	0.00	-
		100	0.004 [0.001, 0.009]	-0.094 [-0.122, -0.067]	0.00	-
		1000	0.001 [0.000, 0.004]	-0.097 [-0.125, -0.07]	0.00	-
	urban area (C)	10	0.097 [0.072, 0.127]	0.000 [-0.018, 0.019]	0.49	-
		100	0.075 [0.055, 0.099]	-0.021 [-0.041, 0.001]	0.03	-
		1000	0.050 [0.033, 0.068]	-0.046 [-0.070, -0.022]	0.00	-
	urban area (R)	10	0.025 [0.013, 0.043]	-0.071 [-0.091, -0.053]	0.00	-
		100	0.009 [0.003, 0.017]	-0.087 [-0.115, -0.066]	0.00	-
		1000	0.007 [0.001, 0.015]	-0.089 [-0.117, -0.067]	0.00	-
	annual mean temperature (C)	10	0.124 [0.102, 0.157]	0.030 [0.007, 0.051]	1.00	19.0
		100	0.122 [0.100, 0.156]	0.028 [0.001, 0.050]	0.97	**49.0**
		1000	0.122 [0.098, 0.155]	0.027 [-0.003, 0.050]	0.96	**49.0**
	annual mean temperature (R)	10	0.075 [0.058, 0.094]	-0.022 [-0.041, -0.003]	0.01	-
		100	0.065 [0.050, 0.082]	-0.033 [-0.053, -0.012]	0.00	-

	1000	0.063 [0.048, 0.080]	-0.034 [-0.054, -0.014]	0.00	-
annual precipitation (C)	10	0.090 [0.072, 0.117]	-0.024 [-0.036, -0.013]	0.00	-
	100	0.045 [0.032, 0.062]	-0.069 [-0.088, -0.053]	0.00	-
	1000	0.034 [0.023, 0.049]	-0.080 [-0.101, -0.064]	0.00	-
annual precipitation (R)	10	0.098 [0.078, 0.123]	-0.015 [-0.022, -0.008]	0.00	-
	100	0.089 [0.071, 0.112]	-0.024 [-0.033, -0.015]	0.00	-
	1000	0.088 [0.070, 0.111]	-0.025 [-0.035, -0.016]	0.00	-

Table S4. Impact of several environmental factors on the dispersal velocity of WNV lineages in North America - a comparison between subsets of lineages (before and after 2002, as well as between WNV genotypes). We here report the mean Q statistics (Q_{mean}) and associated Bayes factors (BF) supports. The results are all based on 100 posterior trees obtained by spatially-explicit phylogeographic inference. "C" and "R" indicate if the considered environmental raster was considered as a conductance ("C") or resistance factor ("R"), and *k* is the rescaling parameter used to transform the initial raster (see the text for further details). BF supports based on the randomisation procedure are only reported when p(Q > 0) is at least 90%. Following Kass & Raftery (1995), we consider a BF value >20 as strong support for a significant correlation between the environmental distances and dispersal durations (in bold).

**Path model**	**Environmental factor**	***k***	**All branches**		**Before 2002**		**After 2002**		**NY99 genotype**		**WN02 genotype**		**SW03 genotype**	
			**Q_m**	**BF**	**Q_m**	**BF**	**Q_m**	**BF**	**Q_m**	**BF**	**Q_m**	**BF**	**Q_m**	**BF**
**Least-cost path model**	elevation (C)	10	-0.004	-	0.004	0.7	-0.001	-	-0.015	-	-0.003	-	-0.003	-
		100	-0.008	-	0.010	0.9	-0.006	-	-0.025	-	-0.004	-	0.000	-
		1000	-0.015	-	0.009	-	-0.014	-	-0.034	-	-0.011	-	0.006	-
	elevation (R)	10	-0.006	-	-0.009	-	-0.010	-	-0.014	-	-0.001	-	-0.006	-
		100	-0.019	-	-0.017	-	-0.031	-	-0.060	-	-0.012	-	-0.011	-
		1000	-0.023	-	-0.02	-	-0.037	-	-0.075	-	-0.017	-	-0.011	-
	forests (C)	10	-0.011	-	-0.009	-	-0.022	-	-0.065	-	-0.011	-	0.005	-
		100	-0.021	-	-0.022	-	-0.036	-	-0.094	-	-0.024	-	0.006	-
		1000	-0.025	-	-0.026	-	-0.041	-	-0.096	-	-0.028	-	0.003	-
	forests (R)	10	0.013	0.3	0.009	0.3	0.015	0.6	0.017	-	0.013	0.3	0.009	1
		100	-0.004	-	0.003	-	-0.016	-	0.010	-	-0.002	-	0.017	-
		1000	-0.011	-	0.000	-	-0.028	-	0.005	-	-0.008	-	0.005	-
	shrublands (C)	10	-0.005	-	0.001	-	-0.007	-	0.001	-	-0.002	-	-0.011	-
		100	-0.005	-	0.001	-	-0.007	-	0.000	-	-0.002	-	-0.013	-
		1000	-0.006	-	0.003	-	-0.005	-	-0.010	-	-0.001	-	-0.013	-
	shrublands (R)	10	0.003	-	-0.006	-	-0.001	-	0.000	-	0.001	-	0.025	10
		100	-0.029	-	-0.025	-	-0.042	-	-0.002	-	-0.033	-	0.019	-
		1000	-0.039	-	-0.029	-	-0.051	-	-0.021	-	-0.049	-	0.016	-
	savannas (C)	10	0.002	-	0.005	-	0.005	-	-0.019	-	0.007	0.6	-0.006	-
		100	0.007	0.3	0.010	0.9	0.008	-	-0.019	-	0.013	0.6	-0.006	-
		1000	0.014	0.4	0.015	0.7	0.007	-	-0.015	-	0.020	0.6	0.004	-
	savannas (R)	10	-0.004	-	0.000	-	-0.001	-	-0.037	-	-0.002	-	0.003	-
		100	-0.045	-	-0.015	-	-0.029	-	-0.075	-	-0.039	-	0.007	-
		1000	-0.057	-	-0.023	-	-0.066	-	-0.081	-	-0.060	-	-0.007	-
	grasslands (C)	10	-0.003	-	0.006	-	0.007	-	-0.002	-	-0.004	-	0.002	-
		100	0.002	-	0.012	0.9	0.016	0.8	-0.040	-	0.004	-	0.011	-
		1000	0.000	-	0.011	-	0.028	4.9	-0.062	-	0.006	-	0.041	8.1
	grasslands (R)	10	-0.017	-	-0.01	-	-0.026	-	0.001	-	-0.013	-	-0.014	-
		100	-0.038	-	-0.024	-	-0.057	-	0.003	-	-0.039	-	-0.022	-
		1000	-0.043	-	-0.027	-	-0.063	-	-0.035	-	-0.044	-	-0.023	-
	croplands (C)	10	-0.010	-	-0.002	-	-0.020	-	0.002	-	-0.007	-	0.0010	-
		100	-0.033	-	-0.018	-	-0.046	-	0.006	-	-0.031	-	-0.008	-
		1000	-0.041	-	-0.025	-	-0.056	-	-0.022	-	-0.042	-	-0.010	-
	croplands (R)	10	-0.005	-	0.005	-	0.008	-	-0.072	-	0.002	-	-0.003	-
		100	-0.018	-	0.005	-	0.004	-	-0.090	-	-0.006	-	-0.008	-
		1000	-0.022	-	0.005	-	0.000	-	-0.092	-	-0.010	-	-0.010	-
	urban area (C)	10	0.000	-	-0.008	-	-0.004	-	-0.024	-	0.002	-	-0.001	-
		100	-0.004	-	-0.016	-	-0.014	-	-0.040	-	0.001	-	-0.004	-
		1000	-0.017	-	-0.024	-	-0.036	-	-0.063	-	-0.010	-	-0.012	-
	urban area (R)	10	0.005	1.8	0.022	8.1	0.005	1.5	0.038	10.1	0.005	1.3	0.004	6.7
		100	-0.006	-	0.098	6.1	-0.022	-	0.029	-	-0.006	-	0.025	7.3
		1000	-0.041	-	0.085	4.6	-0.061	-	-0.025	-	-0.041	-	0.012	-
	annual mean temperature (C)	10	-0.004	-	-0.009	-	0.000	-	-0.027	-	-0.002	-	-0.006	-
		100	-0.003	-	-0.009	-	0.000	-	-0.026	-	0.000	-	-0.007	-
		1000	-0.003	-	-0.009	-	0.000	-	-0.026	-	0.000	-	-0.008	-
	annual mean temperature (R)	10	-0.010	-	-0.001	-	-0.005	-	-0.045	-	-0.016	-	0.025	12
		100	-0.015	-	0.001	-	-0.009	-	-0.048	-	-0.019	-	0.025	6.1
		1000	-0.016	-	0.002	-	-0.009	-	-0.048	-	-0.020	-	0.025	6.1
	annual precipitation (C)	10	-0.001	-	-0.012	-	-0.006	-	-0.033	-	-0.008	-	0.005	-
		100	-0.005	-	-0.015	-	-0.016	-	-0.034	-	-0.012	-	0.004	-
		1000	-0.006	-	-0.015	-	-0.019	-	-0.034	-	-0.013	-	0.003	-
	annual precipitation (R)	10	-0.002	-	-0.004	-	-0.001	-	-0.033	-	-0.011	-	0.008	-
		100	-0.004	-	-0.003	-	-0.002	-	-0.034	-	-0.012	-	0.007	-
		1000	-0.004	-	-0.003	-	-0.002	-	-0.034	-	-0.012	-	0.007	-
**Circuitscape**	elevation (C)	10	-0.034	-	0.007	-	-0.038	-	-0.012	-	-0.032	-	0.006	-

**path model**		100	-0.073	-	-0.023	-	-0.083	-	-0.048	-	-0.072	-	0.013	-
		1000	-0.087	-	-0.052	-	-0.097	-	-0.070	-	-0.086	-	-0.003	-
	elevation (R)	10	0.005	-	-0.033	-	-0.007	-	0.000	-	0.022	**49**	-0.027	-
		100	-0.026	-	-0.077	-	-0.046	-	-0.045	-	0.007	-	-0.045	-
		1000	-0.035	-	-0.086	-	-0.055	-	-0.074	-	-0.001	-	-0.048	-
	forests (C)	10	-0.034	-	-0.028	-	-0.043	-	-0.074	-	-0.036	-	-0.018	-
		100	-0.068	-	-0.083	-	-0.079	-	-0.119	-	-0.068	-	-0.042	-
		1000	-0.078	-	-0.100	-	-0.089	-	-0.126	-	-0.077	-	-0.052	-
	forests (R)	10	-0.019	-	-0.048	-	-0.021	-	0.023	-	-0.010	-	-0.011	-
		100	-0.046	-	-0.074	-	-0.055	-	-0.005	-	-0.036	-	-0.028	-
		1000	-0.052	-	-0.079	-	-0.062	-	-0.016	-	-0.043	-	-0.034	-
	shrublands (C)	10	-0.011	-	0.005	2.6	-0.012	-	0.005	3.2	0.001	-	-0.026	-
		100	-0.013	-	0.007	2.8	-0.015	-	0.017	4.9	0.004	3.5	-0.034	-
		1000	-0.017	-	0.005	-	-0.019	-	0.021	-	0.003	-	-0.039	-
	shrublands (R)	10	-0.061	-	-0.061	-	-0.077	-	-0.006	-	-0.036	-	-0.055	-
		100	-0.090	-	-0.104	-	-0.100	-	-0.033	-	-0.087	-	-0.067	-
		1000	-0.092	-	-0.105	-	-0.102	-	-0.086	-	-0.091	-	-0.068	-
	savannas (C)	10	-0.001	-	0.001	-	-0.008	-	0.023	-	0.003	-	-0.005	-
		100	-0.012	-	-0.003	-	-0.021	-	0.012	-	-0.006	-	-0.007	-
		1000	-0.030	-	-0.014	-	-0.039	-	0.003	-	-0.025	-	-0.006	-
	savannas (R)	10	-0.049	-	-0.047	-	-0.034	-	-0.069	-	-0.044	-	-0.005	-
		100	-0.093	-	-0.091	-	-0.095	-	-0.096	-	-0.088	-	-0.049	-
		1000	-0.096	-	-0.097	-	-0.103	-	-0.099	-	-0.092	-	-0.060	-
	grasslands (C)	10	-0.008	-	0.001	-	0.000	-	0.000	-	-0.005	-	-0.004	-
		100	-0.011	-	-0.017	-	0.005	-	-0.025	-	0.000	-	-0.020	-
		1000	-0.034	-	-0.056	-	-0.019	-	-0.074	-	-0.021	-	-0.019	-
	grasslands (R)	10	-0.035	-	-0.040	-	-0.055	-	-0.005	-	-0.029	-	-0.047	-
		100	-0.077	-	-0.090	-	-0.094	-	-0.036	-	-0.074	-	-0.066	-
		1000	-0.084	-	-0.099	-	-0.099	-	-0.064	-	-0.081	-	-0.069	-
	croplands (C)	10	-0.053	-	-0.030	-	-0.068	-	-0.013	-	-0.052	-	-0.035	-
		100	-0.076	-	-0.059	-	-0.091	-	-0.059	-	-0.070	-	-0.065	-
		1000	-0.088	-	-0.087	-	-0.101	-	-0.083	-	-0.073	-	-0.074	-
	croplands (R)	10	-0.015	-	-0.039	-	-0.002	-	-0.071	-	0.005	-	-0.040	-
		100	-0.036	-	-0.058	-	-0.026	-	-0.094	-	-0.013	-	-0.053	-
		1000	-0.040	-	-0.061	-	-0.032	-	-0.098	-	-0.018	-	-0.055	-
	urban area (C)	10	0.000	-	-0.056	-	0.003	-	-0.036	-	0.01	-	-0.025	-
		100	-0.021	-	-0.080	-	-0.027	-	-0.056	-	-0.005	-	-0.043	-
		1000	-0.046	-	-0.096	-	-0.061	-	-0.083	-	-0.028	-	-0.059	-
	urban area (R)	10	-0.071	-	0.005	-	-0.081	-	-0.033	-	-0.066	-	0.016	-
		100	-0.088	-	-0.018	-	-0.098	-	-0.070	-	-0.082	-	-0.029	-
		1000	-0.090	-	-0.023	-	-0.100	-	-0.076	-	-0.085	-	-0.039	-
	annual mean	10	0.029	19	-0.019	-	0.026	9.0	0.048	6.7	0.040	**32**	-0.010	-
	temperature	100	0.027	**49**	-0.025	-	0.022	**>99**	0.056	11.5	0.040	**>99**	-0.019	-
	(C)	1000	0.026	**49**	-0.026	-	0.021	**>99**	0.056	11.5	0.040	**>99**	-0.020	-
	annual mean	10	-0.021	-	-0.027	-	-0.02	-	-0.056	-	-0.018	-	0.005	-
	temperature	100	-0.032	-	-0.031	-	-0.031	-	-0.064	-	-0.028	-	0.000	-
	(R)	1000	-0.034	-	-0.032	-	-0.032	-	-0.064	-	-0.029	-	-0.001	-
	annual	10	-0.024	-	-0.032	-	-0.028	-	0.010	-	0.001	-	-0.017	-
	precipitation	100	-0.069	-	-0.052	-	-0.078	-	0.014	-	-0.048	-	-0.043	-
	(C)	1000	-0.080	-	-0.057	-	-0.087	-	0.015	-	-0.061	-	-0.048	-
	annual	10	-0.016	-	-0.010	-	0.014	-	-0.019	-	0.014	-	0.015	-
	precipitation	100	-0.024	-	-0.011	-	0.005	-	-0.023	-	0.009	-	0.007	-
	(R)	1000	-0.025	-	-0.011	-	0.004	-	-0.024	-	0.008	-	0.006	-
