# Supplementary material for: Epidemiological hypothesis testing using a phylogeographic and phylodynamic framework
Source: Nat Commun. 2020 Nov 6;11:5620. doi: 10.1038/s41467-020-19122-z (PMC7648063; doi:10.1038/s41467-020-19122-z)
Supplement: Supplementary file 3 — Reporting Summary [file 41467_2020_19122_MOESM3_ESM.pdf]

nature research

Corresponding author(s): Simon Dellicour

Last updated by author(s): Sep 17, 2020

Reporting Summary

Nature Research wishes to improve the reproducibility of the work that we publish. This form provides structure for consistency and transparency in reporting. For further information on Nature Research policies, see our Editorial Policies and the Editorial Policy Checklist.

Statistics

For all statistical analyses, confirm that the following items are present in the figure legend, table legend, main text, or Methods section.

n/a	Confirmed	
☐	☒	The exact sample size (*n*) for each experimental group/condition, given as a discrete number and unit of measurement
☒	☐	A statement on whether measurements were taken from distinct samples or whether the same sample was measured repeatedly
☐	☒	The statistical test(s) used AND whether they are one- or two-sided<br>*Only common tests should be described solely by name; describe more complex techniques in the Methods section.*
☐	☒	A description of all covariates tested
☐	☒	A description of any assumptions or corrections, such as tests of normality and adjustment for multiple comparisons
☐	☒	A full description of the statistical parameters including central tendency (e.g. means) or other basic estimates (e.g. regression coefficient) AND variation (e.g. standard deviation) or associated estimates of uncertainty (e.g. confidence intervals)
☐	☒	For null hypothesis testing, the test statistic (e.g. *F*, *t*, *r*) with confidence intervals, effect sizes, degrees of freedom and *P* value noted<br>*Give P values as exact values whenever suitable.*
☐	☒	For Bayesian analysis, information on the choice of priors and Markov chain Monte Carlo settings
☒	☐	For hierarchical and complex designs, identification of the appropriate level for tests and full reporting of outcomes
☒	☐	Estimates of effect sizes (e.g. Cohen's *d*, Pearson's *r*), indicating how they were calculated

Our web collection on statistics for biologists contains articles on many of the points above.

Software and code

Policy information about availability of computer code

Data collection	No software was used for data collection.
Data analysis	The alignment of genomic sequences was made using the program MAFFT version 7 (https://mafft.cbrc.jp/alignment/server/) and manually edited using the program AliView version 1.26 (https://ormbunkar.se/aliview/). The preliminary phylogenetic analysis was performed using the maximum likelihood method implemented in the program FastTree version 2.1 (http://www.microbesonline.org/fasttree/). Continuous phylogeographic and phylodynamic inferences were performed with the Bayesian methods implemented in the open-source program BEAST version 1.10.4 (https://beast.community/programs) and the BEAGLE library (version 3) to improve computational performance (https://github.com/beagle-dev/beagle-lib). We used the program Tracer version 1.7 (https://beast.community/tracer) to identify the number of sampled trees to discard as burn-in as well as to inspect the convergence and mixing properties of the BEAST outputs. We used the program TreeAnnotator version 1.10.4 (https://beast.community/programs) to obtain the maximum clade credibility tree. Subsequent dispersal statistics estimation and landscape phylogeographic analyses were implemented and performed with R functions available in the open-source package "seraphim" version 1.0 (http://evolve.zoo.ox.ac.uk/Evolve/Seraphim.html). The BEAST XML files of the continuous phylogeographic and skygrid-GLM analyses, as well as the R scripts and related files needed to run all the landscape phylogeographic testing analyses, are available at https://github.com/sdellicour/wnv_north_america (DOI: 10.5281/zenodo.4035938; see also the README file).

For manuscripts utilizing custom algorithms or software that are central to the research but not yet described in published literature, software must be made available to editors and reviewers. We strongly encourage code deposition in a community repository (e.g. GitHub). See the Nature Research guidelines for submitting code & software for further information.

Data

Policy information about availability of data

All manuscripts must include a data availability statement. This statement should provide the following information, where applicable:

- Accession codes, unique identifiers, or web links for publicly available datasets
- A list of figures that have associated raw data
- A description of any restrictions on data availability

BEAST XML files of the continuous phylogeographic and skygrid-GLM analyses are available at https://github.com/sdellicour/wnv_north_america. WNV sequences analysed in the present study were available on GenBank and deposited before November 21, 2017. Accession numbers of selected genomic sequences are listed in the file "WNV_GenBank_accessions_numbers.txt" available on the GitHub repository referenced above. The source of the different raster files used in this study is provided in Table S1. The administrative flyways were obtained from the US Fish and Wildlife Service (USFWS; https://www.fws.gov/birds/management/flyways.php).

Field-specific reporting

Please select the one below that is the best fit for your research. If you are not sure, read the appropriate sections before making your selection.

☐ Life sciences ☐ Behavioural & social sciences ☒ Ecological, evolutionary & environmental sciences

For a reference copy of the document with all sections, see nature.com/documents/nr-reporting-summary-flat.pdf

Ecological, evolutionary & environmental sciences study design

All studies must disclose on these points even when the disclosure is negative.

Study description	In our study, we analysed a comprehensive data set of West Nile virus (WNV) genomes with the objective of unveiling the dispersal and demographic dynamics of the virus in North America. Specifically, (i) we used a continuous phylogeographic approach to reconstruct the dispersal history of WNV on the continent, (ii) we used a first landscape phylogeographic approach to test the impact of environmental factors on the dispersal locations of WNV lineages, (iv) we used a second landscape phylogeographic approach to test the impact of environmental factors on the dispersal velocity of WNV lineages, (v) we used a third landscape phylogeographic approach to test the impact of migratory bird flyways on the dispersal history, and (vi) we used a phylodynamic approach (a "skygrid-GLM" analysis) to test the impact of environmental factors on viral genetic diversity through time.
Research sample	The analysed data set consists in 993 geo-referenced genomic sequences of the West Nile virus sampled in North America.
Sampling strategy	We started by gathering all WNV sequences available on GenBank on the November 20, 2017. We then only selected sequences (i) of at least 10 kb, i.e. covering almost the entire viral genome (~11 kb), and (ii) associated with a sufficiently precise sampling location, i.e. at least an administrative area of level 2. Administrative areas of level 2 are hereafter abbreviated "admin-2" and corresponds to US counties.
Data collection	We downloaded all West Nile virus genomic sequences available on GenBank on the November 20, 2017. We we then only selected sequences of at least 10 kb and associated with known sampling location and sampling time. The resulting alignment was made of 993 geo-referenced genomic sequences.
Timing and spatial scale	Genomic sequences analysed in the study were collected in North America between 1999 and 2007.The spatio-temporal distribution of selected genomic sequences is displayed in Figure S1.
Data exclusions	There was no data exclusion.
Reproducibility	(No experiment was performed)
Randomization	The only group allocation performed in our study concerned the comparison of the dispersal history and dynamics of five different subset of WNV lineages: lineages occurring during (before 2002) and after the expansion phase (after 2002), as well as lineages assigned to each of the three commonly defined WNV genotypes that circulated in North America ("NY99", "WN02", and "SW03"; see Figures S1 and S2). These group allocations were not random: the genotypes were identified on the basis of the WNV database published on Nextstrain (https://nextstrain.org/WNV/NA?c=lineage).
Blinding	Not applicable because we downloaded all genomic sequences of the West Nile virus available on November 20, 2017.

Did the study involve field work? ☐ Yes ☒ No

Reporting for specific materials, systems and methods

We require information from authors about some types of materials, experimental systems and methods used in many studies. Here, indicate whether each material, system or method listed is relevant to your study. If you are not sure if a list item applies to your research, read the appropriate section before selecting a response.

Materials & experimental systems

n/a	Involved in the study
☒	☐ Antibodies
☒	☐ Eukaryotic cell lines
☒	☐ Palaeontology and archaeology
☒	☐ Animals and other organisms
☒	☐ Human research participants
☒	☐ Clinical data
☒	☐ Dual use research of concern

Methods

n/a	Involved in the study
☒	☐ ChIP-seq
☒	☐ Flow cytometry
☒	☐ MRI-based neuroimaging
